# Segregation

**Published:** 1914-02

**Authors:** 


**A Monthly Review of Medicine and Surgery**

EDITOR AND PUBLISHER DR. A. L. BENEDICT, 228 Summer St., corner of Elmwood Ave., Buffalo. (Address for all communications. Please make personal and telephone calls before 1 P. M.)

Third, in age, in the western continent. Independent but supports professional organization. News limited mainly to western N. Y. and Penn.
Subscription $2.00 a year; $3.00 for two years in advance. Changes and errors in address or failure or duplication of delivery, should be reported immediately.

YEARLY VOLUME 69 FEBRUARY, 1914 No. 7

### Segregation

Editorial and original comments on the segregation of prostitutes have, of recent years, been mainly unfavorable. Some indeed have implied that the advocacy of segregation was an indication of immorality. Without going to the extreme of such advocacy, it seems timely to call attention to the fact that the problem of the control of vice still involves many open issues and, among them, the policy of segregation. It has been argued that segregation is a failure because it neither lessens vice nor the risk of general diseases. This charge is unquestionably true. If we consider the matter impartially, it becomes evident that segregation is not at all analogous to quarantine but rather to the commercial tendency to group business houses of the same general nature in approximately limited districts of a city. Segregation will not diminish vice any more than the grouping of department stores will diminish their sales. On the other hand, we cannot see that segregation will have any tendency to increase vice. The lack of analogy to ordinary commercial houses is due partly to the fact that, under ordinary conditions, there will always be certain districts frequented by houses of prostitution and accessible to the public, partly to the fact that the exclusion of such places from other parts of a city will diminish the factors of suggestion and temptation to those not deliberately seeking such places. There are obvious applications of the general law of chance which tend to make the incidence of venereal disease, at some time, almost inevitable in the case of any person, male or female, who indulges promiscuously in sexual intercourse. Segregation will no more make vice safe than the grouping of automobile agencies will remove tendencies to punctures and engine troubles.

Segregation segregates, and that is all that can be expected of it. Is the limitation of houses of ill fame to one district in a city desirable or undesirable? Perhaps this question can be best answered by putting it in a converse and somewhat personal form. Is it desirable or undesirable that there should be business districts to which decent men and women can go or send their children without the offense, danger and liability to scandal inevitable if the same districts also contain houses of ill fame? Is it desirable or undesirable that a permanent resident of a city may be assured of the respectability of a neighborhood in which he makes his home or invests his money? Is it desirable or undesirable that the transient visitor to a city, or one compelled to board away from home, shall be assured reasonable protection against disreputable associates and neighbors?

There is another reason for advocating segregation. Prostitution, for reasons that need not be discussed here, is attended by a high proportion of liability to suicide, homicide and property damage. Is it desirable or undesirable that the consequent demand for relatively high degrees of police supervision should be limited in area?

The essential objection to segregation is that it implies some degree of license and legal recognition of a nefarious traffic. At first thought, it would appear that the law should neither give its protection nor its cognizance, except in a criminal way, to prostitution. But, without entering into details, it may also be argued that it is sound policy to call a spade a spade and not to follow the reputed folly of the ostrich—of which most naturalists exculpate the bird—of blinding ourselves to the existence of an actual fact. Which of these courses is ultimately better for society should determine public policy, rather than theory as to the dignity of the law and the desirability of maintaining high moral ideas, unless they can be reduced from the abstract to the concrete. In other words, is it better for the law to recognize formally an existing evil and to deal with it as it does with other evils, the liquor traffic, for example, or to follow the present policy of occasional raiding, quasi-supervision, and connivance? We have no hesitation in declaring that one of two policies should be consistently carried out. Either the law should recognize existing facts and license, control and segregate prostitution, at the same time affording the prostitute the same protection and freedom from molestation which it gives to the saloon keeper and to every other citizen, or else it should treat as criminal not only prostitution in the ordinary sense but the social evil in any of its manifestations, without regard to social standing, business interest or sex. There is no justice in punishing a person who engages regularly and as a matter of business in a given pursuit and exempting the occasional and amateur perpetrator of the same social offense. Neither is there justice in persecuting the prostitute and letting her patrons go free, nor reversely, in punishing the male and regarding as an innocent and injured party the female of an indecent escapade.

We believe that the real social evil of the present is not prostitution but the honeycombing of supposedly decent society with essentially the same vice. If, without hypocrisy and with substantial realization of theory in fact, prostitution can be stamped out, the discussion of segregation, license and every other open question in regard to the social evil, should lead to but one conclusion. If, on the other hand, the refusal to recognize, license and segregate will result—or is resulting—in the scattering of the evil where it cannot be controlled and where, after the analogy of scattering of fomites of infection, it will increase social disease, and ultimately produce a social pyæmia like that which killed the Roman Empire, we should be inclined to stand by the judgment of mankind for the last fifty centuries or so and not by that of a modern school of reformers, and should further be inclined to advocate the adaptation of this judgment to modern legal conceptions of the necessity of regulating everything liable to abuse.

